# Acute Traumatic Tetanus Treated by Magnesium Sulphate, with Report of a Case in the Treatment of Which Injections of an Aqueous Twenty-five per Cent Solution of Magnesium Sulphate Were Made in the Spinal Subarachnoid Space; with Recovery

**Published:** 1909-06

**Authors:** Aime Paul Heineck

**Affiliations:** Chicago, Ill.; Professor of Surgery, Reliance Medical College; Adjunct Professor of Surgery, University of Illinois; Surgeon to the Cook County Hospital


THE
### TEXAS MEDICAL JOURNAL.
Established July, 1885.

F. E. DANIEL, M. D., - - - - Editor, Publisher and Proprietor

PUBLISHED MONTHLY.—SUBSCRIPTION $1.00 A YEAR.

VOL. XXIV. AUSTIN, JUNE, 1909. No. 12.

The publisher is not responsible for the views of contributors.

### Original Articles.

For Texas Medical Journal.

### Acute Traumatic Tetanus Treated by Magnesium Sulphate, With Report of a Case in the Treatment of Which Injections of an Aqueous Twenty-five Per Cent Solution of Magnesium Sulphate Were Made in the Spinal Subarachnoid Space; With Recovery.

BY AIME PAUL HEINECK, CHICAGO, ILL.,
Professor of Surgery, Reliance Medical College; Adjunct Professor of Surgery, University of Illinois; Surgeon to the Cook County Hospital.

Our knowledge concerning this acute infectious disease is incomplete. Numerous are the features of this intoxication that call for elucidation. We know that the disease occurs sporadically, endemically (1), and epidemically; that there is no age, sex or race that is immune. It has occurred in Iceland. It is very prevalent in the tropics. In reference to race incidence, it must be stated that it is considered by most observers to be more frequent in the dark-skinned races than in the white race, even in the same country. The disease has a variable period of incubation; on an average, in the acute form, from five to ten days elapse between inoculation and the appearance of the symptom-complex of this condition. A short period of incubation implies intensity and virulency of infection, and is of bad prognostic omen. Though it is not believed that one attack confers immunity against other attacks, cases of second attacks are not known (7).

Though this disease is comparatively rare, it occurs in such unforeseen (8) conditions, and usually has such a dramatic outbreak and such a fatal termination, that it is of interest to all medical practitioners. It has complicated burns (2). It has complicated frostbites. It has complicated horse bites. It has followed such insignificant trauma as is associated with the hypodermic injections of quinine (3), with the subcutaneous administration of anti-plague serum (4), with the application, for hemostatic purposes, of gelatine to bleeding surfaces, with the subcutaneous employment, for hemostatic or other purposes, of this same agent (5), with the operation of vaccination (6), of circumcision of the removal of adenoids. It has followed the employment, in operative procedures, of contaminated catgut, it has followed contused wounds of the outer canthus of the eye (9), and other wounds so insignificant that at the time of infliction they passed unnoticed, or, if noticed, they were completely forgotten at the time of the outbreak of the disease. The disease may occur after childbirth, and may occur after abortion, accidental or induced (10). As a result of Fourth of July injuries in 1903, there were 406 deaths from tetanus as compared with 60 from other sources (11).

Since the discovery by Nicolaier, in 1885, of the bacillus tetani, and its growth, in pure cultures, by Kitasato, in 1889, it has been amply demonstrated that all clinical forms of tetanus, cephalic tetanus (12), tetanus neonatorum (13), puerperal tetanus (14), post-operative tetanus (15), traumatic tetanus, are due to the bacillus tetani. The inoculation of the offending germ occurs through an abrasion or through a wound of a cutaneous, or a mucous surface. Tetanus is an implantation infection. In the lower animals, all experimental efforts to produce the disease, through either the respiratory or the alimentary tract, have proved unsuccessful. In man, as far as we know, the same condition obtains. No case is on record of the disease occurring in man, as a result of infection taking place by inhalation or ingestion of the tetanus bacilli. The bacillus, though not a pyogenetic germ, is not hindered in its development by the presence of the germs of suppuration. The latter, in fact, create conditions favorable for its growth (16). As a wound complication, the frequency of tetanus has markedly lessened since the generalization of the antiseptic treatment of wounds.

The disease has no characteristic pathological anatomical changes (that is, none have to this date been determined, or rather, demonstrated). No constant changes have been found either in the peripheral nerves or in the cerebro-spinal nervous system.

The diagnosis offers no difficulties. In all forms of the disease, the chronic cephalic form excepted, the mortality is appalling. In an editorial in the *Journal of the American Medical Association* (16a) it is stated that "the usual rate of mortality for traumatic tetanus is probably about 80 per cent." Stewart (17) says that "the mortality is greatest in the puerperal type, extremely few cases recovering. It is said that recovery is almost unknown in tetanus after abortion." This high mortality is due to the fact that the measures actually employed in the treatment of this disease are ineffective. It is notorious that the drug treatment of this disease has been without efficacy. Many are the medicinal agents that have been employed in the treatment of tetanus. The indication for their employment has been found chiefly in the controlling or depressing influence which they exert upon muscular action. Opium (18), carbolic acid (19), physostigmine (20), the bromides and chloral hydrate (21) can be mentioned among the drugs that have been, and still are, employed extensively in the treatment of this disease. These drugs meet, more or less successfully, isolated symptoms of this disease. Recoveries from tetanus infection are reported in which the medical attendants attribute the happy termination of the disease to the employment of one or more of the aforementioned drugs. Apparently, none of these drugs exercise much influence upon the course of severe cases. In 1904 J. B. Murphy reported a case of tetanus successfully treated by the intraspinal injection of solution of eucaine B and morphine. Very mild cases recover with, perhaps despite, any of the various forms of treatment.

For prophylactic and for curative purposes, antitetanic serum in liquid or solid form is widely employed. Different routes are employed to introduce the liquid serum into the human organism. The injections of the serum may be subcutaneous, intramuscular (21a), intravenous (22), intraneural (23), intracerebral (24) and (30a, Girard), or intraspinal (25). In the intraspinal method, some clinicians introduce the antitetanine in the epidural space (26); the majority, however, make the injection in the spinal subarachnoid space. In all wounds of a suspicious nature, such as those in which there is much contusion of tissue, such as are soiled with street-dirt or garden-earth, in all gunshot wounds, in wounds occurring in individuals who work around horses, in horseshoeing establishments, or in stables, it is the practice of most surgeons to inject for prophylactic purposes, in the wounded individual, from 2000 to 3000 units of antitetanic serum. The sooner after the injury the serum is injected, the greater is its protective power, the greater is its prophylactic potency. For the last ten years, in all individuals having wounds of the nature described above, I have injected for prophylactic purposes, invariably, antitetanic serum. I have never seen a case of tetanus occur after attempted immunization. It must be stated, however, that, lately, the immunizing properties of antitetanic serum have been disputed. Some cases of tetanus have been reported which show that antitetanic serum is not invariably successful in preventing the outbreak of the disease. Jacobson and Pease (21a) were able to collect six cases occurring in the United States and Canada, in which, despite the previous prophylactic use of antitetanic serum, tetanus developed. In all but one of these cases, recovery ensued. Reynier (27) was able to collect from the literature thirty-one other cases of tetanus that had developed subsequently to attempted immunization by prophylactic injections of antitetanic serum. To these, he added one personal case. In this series, though the antitetanic serum did not prevent the disease, it, apparently, in most of the cases, attenuated the symptoms and positively lessened the mortality rate. Mauclaire, *Gazette des Hôpitaux,* 1903, No. 43, p. 439, reports a case of tetanus consecutive to a fracture of both bones of the forearm, due to a horse bite. A prophylactic injection of antitetanic serum was administered, but, nevertheless, the disease developed. It was an attenuated form of the disease. It lasted twenty-five days; treatment, antitetanic serum and chloral. Recovery, in the lower animals, the immunizing properties of antitetanic serum have been repeatedly demonstrated. In laboratory experiments, the serum being usually injected either simultaneously with, or immediately after, the injection of the toxin, neutralization is easily effected and tetanus does not develop. Owing to the employment, as a preventive of tetanus, of antitetanic serum, by veterinarians, this disease as a wound complication after castration of horses has almost completely disappeared. In the human subject, the immunizing properties of antitetanic serum are not as universally acknowledged.

As in immunizing doses, antitetanic serum is perfectly innocuous, we urge, until more light be thrown on the subject, that it be employed as a prophylatic agent against tetanus. Schwartz (30a) in 300 injections noticed no other accident but an occasional erythema (five cases). In the opinion of many clinicians, its value as a preventive of the disease is established (30). Delbet, Demoulin (27), and Kummer (28), and innumerable other observers, have never seen tetanus develop in a patient to whom, shortly after the infliction of his injury, an immunizing dose of antitetanic serum had been administered. It must be stated, however, that the value of antitetanic serum, as a prophylactic agent, is based on belief, on clinical observation, and not on scientifically demonstrated facts. In the Paris hospitals (27) prophylactic injections of antitetanic serum were not employed between the years of 1886-1890, inclusive. During this period there were in the city of Paris 135 deaths from tetanus. During the years 1901-1905, inclusive, the prophylactic injections were employed in nearly all, if not all, the Parisian hospitals. The serum during this same period was also extensively employed as a curative agent. During the years 1901-1905, inclusive, there occurred in Paris 153 deaths from tetanus.

In the prophylactic treatment of tetanus, in addition to the administration of antitetanic serum, all suspicious (suspicious from the standpoint of tetanus development) wounds should be subjected to vigorous and thorough antiseptic treatment. Lowering of vitality by bruising, and incorporation of foreign material, favor but are not essential for the development of tetanus. Like all sporulated microbes, the bacillus of Nicolaier offers great resistance to the action of antiseptics.

The following table is taken from an article by Scherck (29). It constitutes quite a forcible plea for the prophylactic employment of antitetanic serum:

Cases of Fourth of July injuries treated in the city dispensaries of St. Louis:

| Years. | Number case. | Antitetanic serum. | Death from tetanus. |
|---|---|---|---|
| 1903 | 56 | No. | 16 |
| 1904 | 37 | Yes. | None. |
| 1905 | 84 | Yes. | None. |
| 1906 | 170 | Yes. | None. |

In the treatment of numerous cases of tetanus occurring in the human subject, antitetanic serum has been employed. In many cases thus treated, recovery ensued. It is conceded, however, that in the great majority of cases in which this agent has been used, whatever may have been the route of introduction of the serum into the human system, the results have been disappointing. The cases have terminated fatally, not on account of the administration of antitetanic serum, but because of the inefficacy of the latter as a curative agent for tetanus. So extremely unsatisfactory have been the results attending its use, that though still extensively employed, it is regarded as inefficacious by all, being employed for want of a better agent. The serum exerts but little influence on the course of the malady, and, despite its use, the large majority of cases result in death.

Jacobson and Pease (21a) say: "It is apparent that after tetanus is fully established, serum therapy, however administered, promises but little as a curative agent." In a discussion before the Société de Chirurgie de Paris (27), in which most of those present participated, the opinion was general that, as a curative agent for tetanus, antitetanic serum in the human subject is of doubtful efficacy. Calmette, himself, expresses the opinion that antitetanic serum has no curative power, but that, in chronic tetanus, it markedly shortens the duration of the illness. The report of a case, in which a comparatively new mode of treatment has been employed with success, finds its justification in the fact that in the present state of our knowledge all forms of treatment, in this disease, are extremely unsatisfactory.

Mr. Otto Copeck, 17 years of age, Bohemian by birth, was admitted to the West Side Hospital on October 22, 1908. Eight days previous to admission he had stepped upon an old, rusty horseshoe nail, thereby sustaining a punctured wound of the left foot. Though no attempt at disinfection had been made, this punctured wound, about an inch in depth, had by the time of admission, healed by first intention. Two days before admission, patient suffered from general malaise. On October 21st, neck began to feel stiff and sore, and patient began to experience some difficulty in opening his mouth. On the morning of October 22d, Dr. Vasumpaur was called, examined the patient, and made a diagnosis of acute traumatic tetanus. He gave a subcutaneous injection of 2500 units of antitetanic serum, and ordered that an ambulance be called, and that the patient be conveyed to the hospital and placed under my care. When I first saw the case, the manifestations of the disease were so classical that the diagnosis of tetanus was self-evident. There were present trismus, retraction of the head, marked rigidity of the cervical, thoracic, and abdominal muscles, opisthotonos, etc. The angles of the mouth were drawn outward and downward, the upper lip firmly pressed against the teeth, producing the facial expression which is almost invariably present in this disease. The voice was feeble. Slight disturbance of the patient, as by loud talking, opening and closure of the door, etc., would excite convulsive seizures of about ten seconds' duration. The patient remained in the hospital twenty-eight days. The period of convalescence began on the tenth day after admission to the hospital and was uneventful. His treatment after the first ten days consisted merely of careful nursing. During the first eight days of the active stage of the disease, patient suffered from retention of the urine. The application of fomentations to the hypogastrium having failed to relieve the condition, he was catheterized three times daily from October 22d to November 2d. No vesical disturbance resulted. During this same period patient was obstinately constipated. Cathartics per mouth and rectal enemata being without influence, resort was had to the subcutaneous administration of physostigmine salicylate in doses of grain 1-100, and relief was thereby obtained. In the acute stage of the disease, two such doses were taken. In the first few days, attempts to give enemata would provoke convulsive seizures.

From October 22d to November 2d, inclusive, patient's diet was wholly liquid. On the evening of November 6th, he was started on semi-solid food. On the 19th of November he was discharged. During the active stage of his illness, our patient received, to combat insomnia, an occasional dose of morphine. On admission into the hospital, 4500 units of antitetanic serum were injected in the spinal subarachnoid space, 1500 units subcutaneously around the left sciatic nerve, just beneath the gluteal fold, 1500 units in the region of the anterior crural nerve, about an inch below Poupart's ligament. On October 23d, 7500 units of serum were injected subcutaneously. On October 24th, 6000 units were introduced in the spinal subarachnoid space. On October 25th, 6000 units were injected in the subarachnoid space, 1500 units in the left foot, in the region of the wound of inoculation, and the same amount around the left sciatic nerve. On October 26th, 6000 units were injected in the subarachnoid space, and 1500 units subcutaneously around the left sciatic nerve. On October 28th, 4500 units were given subarachnoidally, 1500 units in the left sciatic nerve, and 1500 units in the left foot. On October 30th, again 6000 units were injected into the spinal subarachnoid space, and 3000 units subcutaneously.

All the injections in the subarachnoid space were made either through the interspace between the spinous processes of the third and fourth lumbar vertebræ, or through that between the fourth and fifth lumbar vertebræ. For these injections, as well as for those of the aqueous solution of magnesium sulphate, anesthesia was not used. Anesthesia is not necessary. General anesthesia is decidedly harmful in these cases. It has determined deaths. Five injections, each of 5 c.c., of an aqueous 25 per cent solution of magnesium sulphate, were introduced into the spinal subarachnoid space. The path of injection was the interspace between the spinous processes of the fourth and fifth lumbar vertebræ. The needle was inserted about 2 cm. to the side of the median line, on a level with an imaginary line extending between the highest point of each iliac crest. None of the solution was injected until a few drops of clear nonblood-stained cerebro-spinal fluid had escaped.

The magnesium sulphate injections were made on the 23d, 25th, 26th, 28th and 30th of October. Each injection was followed by marked lessening of muscular rigidity and noticeable improvement in the patient's general condition. Upon reappearance of the symptoms to an extreme degree, the injections would be repeated. After the first injection, the rigidity of the lower limbs never returned to any but a slight degree. I can not but be of the opinion that the magnesium sulphate was a contributory factor to the patient's recovery.

Previous to our employment of magnesium sulphate, it had been used by other clinicians. Their cases follow. In some of these cases, death occurred; in others, recovery followed. The cases as yet are too few in number for any definite opinion to be expressed as to its value. A more exact dosage must be determined. Greater proficiency in administering must be obtained. The results, however, have been sufficiently encouraging to warrant, in fact, to demand, further study of the subject. The experimental work on this subject has been done chiefly, almost wholly, by Meltzer and Auer (31). They determined that intraspinal injections of magnesium salts are capable of abolishing completely in monkeys, at least temporarily, both tonic and clonic tetanic contractions. Clinically, experience seems to partially bear out the further statement of these investigators that intraspinal injections of magnesium sulphate in doses which do not affect the respiratory center or other vital functions, are capable of abolishing completely all clonic convulsions and tonic contractions in cases of tetanus, occurring in the human subject. The relaxing effects of the injections may last twenty-four hours or longer. In the case which I report, none of the vital functions were influenced by the intraspinal injections of magnesium sulphate. In some parts of the body, such as in the lower extremities, the muscular relaxation following upon the injections was complete. In other portions, such as the mandibular, facial, or cervical muscles, the rigidity was very much lessened, but it was not completely overcome. Was it due to insufficient dosage, I am unable to state. Appended to the article is a temperature, pulse, and respiratory chart, in the perusal of which it will be seen that the injections at times were followed by an elevation of temperature. This has been noted by other observers. In Miller's (33) case, the injections determined a profuse secretion of mucus, bronchorrhea, at times severe enough to embarrass respiration, but easily controlled by atropine. Was there a relation of cause and effect between the injections and the elevation of temperature? This must also be decided by further study of the subject. Meltzer and Auer (32) have determined that when administered by the intravenous route, the magnesium salts are very toxic, and that even small doses completely inhibit the respiration. Therefore, for the administration of these salts, this route, the intravenous route, should never be employed. We employed the agent only in the shape of injections in the spinal subarachnoid space.

In all of the tabulated cases, the magnesium sulphate was injected in the subarachnoid space. The solution has also been used subcutaneously in the following three cases:

Lyon (35) reports the following case: Male, 7 years, stepped on a nail which entered the left foot after perforating sole of his shoe. It barely penetrated the skin. Wound scarcely noticeable. Eight days later, complained of stiffness of foot and of leg. Convulsions on the ninth day. On the eleventh day, the jaws were set and almost all of his muscles were rigid. The wound was opened and treated with peroxide of hydrogen and tincture of iodine. Morphine, chloral and bromides partially controlled the convulsions. On the twelfth day, two drachms of magnesium sulphate in 4 oz. of distilled water, were injected under the skin of the abdomen. At end of two hours, jaws could be opened 2 cm. Muscles were markedly relaxed. On the thirteenth, fourteenth, seventeenth and nineteenth days, the magnesium sulphate injection was repeated. The convulsions had become infrequent and mild. Twice, there was bronchorrhea. A vesicular eruption covering the whole body appeared on the fourteenth day. The vesicles were pin-head size and were filled with a clear fluid. In a week, these dried up and disappeared with exfoliation of the epidermis. Digitalis necessary to improve heart action after first week. During the patient's convalescence, tonics were given for the anemia. Able to sit up on the thirtieth day. Walked as usual in about ten days more.

Greeley (36) employed, with success, magnesium sulphate in aqueous solution in two cases of tetanus. As his mode of administration was the subcutaneous, we will briefly mention and not discuss them. The first case occurred in a boy, 2 years old. The child had stepped on an old garden rake and lacerated the web between the great and the adjoining toe of the left foot. After an incubation period of ten days, the symptoms appeared. Greeley administered 7500 units of antitetanic serum. In addition, every two hours, 5 grains each of chloral hydrate and of potassium bromide were administered. By hypodermoclysis, one pint of distilled water containing 2 drachms of magnesium sulphate were introduced into the organism. This was repeated on the next day. Recovery followed.

Greeley's other case was one of chronic tetanus. Four weeks elapsed between the inoculation and the outbreak of the symptoms. By hypodermoclysis, 3 drachms of magnesium sulphate dissolved in a pint of distilled water were introduced into the organism. Recovery ensued.

Wm. Hessert (34) a few weeks ago showed to the Chicago Medical Society a case of acute tetanus successfully treated with subarachnoidean injections of an aqueous 25 per cent solution of magnesium sulphate.

We can not, and we are unwilling to, make any statement as to the value of magnesium sulphate as a therapeutic agent in the treatment of tetanus. The cases in which this agent has been used are, as yet, too few in number to allow the expression of an authoritative opinion. Further laboratory experiments and numerous clinical reports are needed. The animal experiments conducted by Cruveilhier (37) are too few to be conclusive. His findings are contradicted by clinical observers. We would refer the reader to appended tables. The faith which Cruveilhier reposes in antitetanic serum as a curative agent is not warranted by the results that this agent has yielded.

We used magnesium sulphate, in the method stated above, in our case, and the results were so surprising and so satisfactory that we feel justified in urging its use in tetanus. It is important that the utility and the value of this drugs as an agent to control the tonic and clonic muscular contractions so characteristic of this disease be exactly determined. Its value must be decided by the combined experience of clinicians the world over.

[NOTE.—Bibliography omitted for want of room.—ED.]

**Cases of Tetanus in the Treatment of Which Subarachnoid Injections of an Aqueous Solution of Magnesium Sulphate Have Been Employed.**

| | Sex, Age, Weight. | Period of Incubation; Previous Immunization; Nature of Wound. | Other Treatment. | Magnesium Sulphate Treatment. | Result. | Comments. |
|---|---|---|---|---|---|---|
| 1. Blake, Jos. A.—The use of magnesium sulphate in the production of anesthesia and in the treatment of tetanus. Surgery, Gynecol., and Obst., Chicago, 1906, vol. ii, p. 541. | Male, 15 years, 115 pounds. | Seven days. None. Crushed first three fingers of left hand. | Antiseptic disinfection of wound. On third day of disease (tenth of injury) 40 cm. of antitetanic serum injected in spinal cord between fourth and fifth cervical vertebrae; 20 c.c. injected in median cephalic vein. On night of same day 20 c.c. injected in median basilic vein. On eleventh day after injury, 35 c.c. of antitetanic serum injected in spinal canal by lumbar puncture. Chloral hydrate and morphine given when patient not under the effect of magnesium sulphate. | On twelfth day after injury intraspinal injection of 4.5 c.c. of magnesium sulphate (25 in 100 of water). Thirty-three hours later repeated injection. Thirty-seven and one-half hours later intraspinal injection, 8 c.c. of a 12½ per cent solution of magnesium sulphate. Twenty-seven hours later repeated above injection. Six days after repeated same injection. | Recovery. | Injections have a marked effect in restraining the convulsions and relieving pain, thereby conserving strength and preventing excessive metabolism and heat production. |
| 2. Markoe, F. H.—Reference same as case 1, p. 549. | Male, 4 years, 40 pounds. | Seven days. None. Sloughing wound of skin and subcutaneous tissue of the right leg. | Four injections each of 5 c.c. of antitetanic serum were injected into buttock, the external jugular vein, the spinal canal, and back, respectively. Occasional doses of morphine and chloral. | 1.5 c.c. of a 25 per cent solution of magnesium sulphate were slowly injected into the subarachnoid space. | Died twenty-eight hours after the first symptom of disease appeared. | Death cannot be attributed in the slightest degree to the magnesium sulphate. On autopsy cultures of tetanus bacillus were obtained from the wound, spleen, and heart blood, showing a marked tetanus bacteriemia. |

**HEINECK: ACUTE TRAUMATIC TETANUS—continued.**

**Cases of Tetanus in the Treatment of Which Subarachnoid Injections of an Aqueous Solution of Magnesium Sulphate Have Been Employed.**

| | Sex, Age, Weight. | Period of Incubation; Previous Immunization; Nature of Wound. | Other Treatment. | Magnesium Sulphate Treatment. | Result. | Comments. |
|---|---|---|---|---|---|---|
| 3. Logan, Samuel—The treatment of tetanus by intraspinal injections of magnesium sulphate for the control of convulsions. Jour. A. M. A. 1906, vol. xlvi, p. 1502. | Male, 11 years, 80 pounds. | Eight days. None. Gunshot wound of hand with old toy pistol loaded with blank cartridge. | Simple cleansing of wound after development of the disease. On day of admission 50 c.c. of antitetanic serum injected intraspinally. Chloral hydrate, gr. 15; sodium bromide, gr. 30, every four hours. On third day after admission 10 c.c. antitetanic serum injected in each brachial plexus, in each sciatic nerve, and into the tissues around wound; in all 50 c.c. | On third day after admission general anesthesia. 4 c.c. of a 25 per cent solution of magnesium sulphate injected in spinal canal by lumbar puncture. On fourth day again gave patient general anesthesia and injection in subarachnoid space by lumbar puncture 50 minims of 25 per cent solution magnesium sulphate. | Death forty hours and fifty minutes after first injection of magnesium sulphate. Heart failed before respirations affected. | Temperature post-mortem 108.2 F., per rectum. Complete cessation of muscular convulsions following introduction of magnesium sulphate. |
| 4. Logan, Samuel—Reference same as above. | Female, 24 years. | Seventeen days. None. Vaccination. | 100 c.c. of antitetanic serum injected subcutaneously. Thirty hours after appearances of first symptom, wide excision of vaccination wound and dusting of surface with dried antitetanic serum. | Thirty hours after first symptoms were noticed 4 c.c. of a sterile 25 per cent solution of magnesium sulphate were injected into spinal subarachnoid space by lumbar puncture. Local anesthetic employed. Seventeen and one-half hours later, injection was repeated. | Death fifty hours after appearance of first symptoms. | No good resulted from the use of the magnesium sulphate solution. Patient was moribund when second injection of magnesium sulphate was made. |

**HEINECK: ACUTE TRAUMATIC TETANUS—continued.**

**Cases of Tetanus in the Treatment of Which Subarachnoid Injections of an Aqueous Solution of Magnesium Sulphate Have Been Employed.**

| | Sex, Age, Weight. | Period of Incubation; Previous Immunization; Nature of Wound. | Other Treatment. | Magnesium Sulphate Treatment. | Result. | Comments. |
|---|---|---|---|---|---|---|
| 5. Franke, Margan. Ein Fall von Tetanus behandelt mit intraduralem Injectionen von Magnesium Sulphuricum. Zentral. fuer Innere Medicin, 1907, vol. xxviii, p. 344. | Male, 32 years. | Twelve days. None. Wound of the middle finger. | Energetic antiseptic handling of wound is recommended by this author. Amputation of finger. Chloral hydrate, gr. 30, per rectum daily. | Nineteen days after infliction of injury, intradural injection of 1 c.c. of sterilized 25 per cent solution of magnesium sulphate. Five days after above intradural injection of 2 c.c. of same solution. Four days later repeated same injection. Injecting needle broke in tissues. Removed by operation. | Recovery. | Franke noticed after each injection of magnesium sulphate that there was a lessening of contracture, also noticed that the injections exerted a beneficial action on the muscular convulsions. Sleep was better. Nourishment possible. |
| 6. Robinson, G. Canby—Treatment of tetanus by intraspinal injections of magnesium sulphate. Jour. Am. Med. Assn., 1907, vol. xlix, p. 493. | Male, 11 years, 67½ pounds. | Contusion of scalp. None. Played considerably around stable. | Excised supposed wound of entrance. Chloral hydrate, gr. 30; sodium bromide, gr. 60, every twenty-four hours for the first two weeks. | On the eleventh day of the disease, patient was anesthetized. Ethyl chloride used as a general anesthetic. 3 c.c. of a 25 per cent solution of magnesium sulphate injected in subarachnoid space. On the next day repeated injection using 3½ c.c. On fifteenth day of disease injected in same locality 4 c.c. of same solution. | Recovery. | Author states that the intraspinal injections of magnesium sulphate produced marked lessening of the very severe symptoms for a number of hours. The muscular rigidity was never so severe after each injection as it had been before. |

**Cases of Tetanus in the Treatment of Which Subarachnoid Injections of an Aqueous Solution of Magnesium Sulphate Have Been Employed.**

| | Sex, Age, Weight. | Period of Incubation; Previous Immunization; Nature of Wound. | Other Treatment. | Magnesium Sulphate Treatment. | Result. | Comments. |
|---|---|---|---|---|---|---|
| 7. Metzer, S. J., and Auer, Jno.—The Journal of Experimental Medicine, 1906, vol. vii, p. 705. | Male, 35 years. | Four days. Insignificant wound of foot, which healed rapidly. | Large doses of antitetanic serum and sedatives gave no relief. Two hours before death, an intravenous injection of antitoxine serum was given. | One intraspinal injection of magnesium sulphate, 1 c.c. to every eighteen pounds of body weight. | Death five hours after injection of magnesium sulphate solution in subarachnoid space. | Anesthetizing and relaxing effect complete. Respiration good to end. |
| 8. Miller, Robert T.—Treatment of tetanus with subarachnoid injections of magnesium sulphate. The Am. Jour. of the Med. Sciences, 1908, vol. cxxxvi, p. 781. | Male, 7 years, 60 pounds. | Seven days. None. Lacerated wound of left hand. | Antitoxin daily for fourteen doses varying from 1,500 to 7,000 units. Sedatives for a short time. Copious saline enemas and infusion. | Eleven lumbar punctures made within thirteen days. Approximately 2.5 c.c. of a 25 per cent solution of magnesium sulphate being injected into the meninges at each puncture. | Recovery. | "Of the value of the treatment by magnesium sulphate, no one who witnessed this case has any doubt." The muscular paralysis following each injection lasted from eighteen to twenty-nine hours. It involved all muscles, except those of head, neck, and diaphragm. The injections were followed several times by respiratory collapse lasting eleven to fourteen hours and the pulse dropped, though not to a dangerous degree. |
| 9. Henry, Jno. Norman—International Clinics, 1908, Series 18, vol. lv, p. 1. | Male, 9 years. | Six weeks. None. Abrasion of skin of back by kick of horse. | | Lumbar puncture 3 c.c. of 25 per cent solution of magnesium sulphate injected in subarachnoid space. Five days later subarachnoid injection repeated. | Recovery. | The case was a severe one. Made an excellent recovery. Each injection was followed by a relaxation of the rigidity. |

### HEINECK: ACUTE TRAUMATIC TETANUS—continued.

**Cases of Tetanus in the Treatment of Which Subarachnoid Injections of an Aqueous Solution of Magnesium Sulphate Have Been Employed.**

| | Sex, Age, Weight. | Period of Incubation; Previous Immunization; Nature of Wound. | Other Treatment. | Magnesium Sulphate Treatment. | Result. | Comments. |
|---|---|---|---|---|---|---|
| Case II. | Male, 19 years, 123½ pounds. | Seven days. None. Stepped on a nail. At time of admission, the wound was healed. | Wound of foot excised. | Lumbar puncture 6 c.c. of sterile solution of magnesium sulphate injected into spinal canal. Ethyl chloride used as anesthetic. | Death. Admitted July 30th, died August 2nd. | One hour injection patient was entirely relaxed. A rise of temperature followed the intraspinal injection. |
| Case III. | Male, colored, 9 years, 55 pounds. | Six days. None. Stepped on nails with both feet and inflicted punctured wounds | | Lumbar puncture, 4 c.c. of clear spinal fluid withdrawn. 2½ c.c. of 25 per cent solution of magnesium sulphate injected into spinal canal. Two days later repeated injection, only gave 2 c.c. at second injection. | Death. | A rise of temperature followed each injection. |
| Case IV. | Male, 45 years. | Three weeks. None. Stepped on nail. | On same day as second subarachnoid injection, 18 c.c of antitetanic serum were given subcutaneously. On the morrow, 30 c.c. of antitetanic serum were injected into the left buttock. | 6 c.c. of 25 per cent solution of magnesium sulphate injected into subarachnoid space by lumbar puncture. Three days after above, performed lumbar puncture, removed 35 c.c. of clear spinal fluid, and injected 6 c.c. of solution of magnesium sulphate. | Death on evening of second day following second injection. | "It is very much a question whether the magnesium sulphate did not contribute to the patient's death." |

